# How does the increase in eating difficulties according to the Development and Well‐Being Assessment screening items relate to the population prevalence of eating disorders? An analysis of the 2017 Mental Health in Children and Young People survey

**DOI:** 10.1002/eat.23833

**Published:** 2022-10-20

**Authors:** Jessica O'Logbon, Tamsin Newlove‐Delgado, Sally McManus, Frances Mathews, Suzanne Hill, Katharine Sadler, Tamsin Ford

**Affiliations:** ^1^ Department of Psychiatry University of Cambridge Cambridge UK; ^2^ Medical School University of Exeter Exeter UK; ^3^ National Centre for Social Research London UK

**Keywords:** eating behavior, eating disorder, prevalence, screening, survey

## Abstract

**Objective:**

We examine the test accuracy of the Development and Well‐Being Assessment (DAWBA) eating disorder screening items to explore whether the increased eating difficulties detected in the English National Mental Health of Children and Young People (MHCYP) Surveys 2021 reflect an increased population prevalence.

**Methods:**

Study 1 calculated sensitivity, specificity, and positive and negative predictive values from responses to the DAWBA screening items from 4057 11–19‐year‐olds and their parents, in the 2017 MHCYP survey. Study 2 applied the positive predictive value to data from 1844 11–19‐year‐olds responding to the 2021 follow‐up to estimate the prevalence of eating disorders in England compared to 2017 prevalence.

**Results:**

Parental report most accurately predicted an eating disorder (93.6%, 95% confidence interval: 92.7–94.5). Sensitivity increased when parent and child answers were combined, and with a higher threshold (of two) for children. The prevalence of eating disorders in 2021 was 1% in 17–19‐year‐olds, and .6% in 11–16‐year‐olds—similar to the prevalence reported in 2017 (.8% and .6%, respectively). However, estimates for boys (.2%–.4%) and young men (.0%–.4%) increased.

**Discussion:**

We found tentative evidence of increased population prevalence of eating disorders, particularly among young men. Despite this, the DAWBA screening items are useful for ruling *out* eating disorders, particularly when parents or carers screen negative, but are relatively poor at predicting who will have a disorder. Data from both parents and children and applying a higher cut point improves accuracy but at the expense of more missed cases.

**Public Significance Statement:**

The prevalence of eating disorders did not markedly change from 2017 to 2021, but we found tentative evidence of an increase, particularly among young men. This is despite larger increases in problematic eating, which need further investigation. The DAWBA screen is best suited to ruling out eating disorders which limits its clinical applications as it would provide many false positives requiring further assessment.

## 1 INTRODUCTION

The prevalence of eating disorders peaks during adolescence (Potterton et al., 2020)—a life stage associated with important milestones and transitions, and during which impaired functioning can catastrophically undermine subsequent health, educational, and social outcomes. Eating disorders are pernicious, multi‐factorial conditions whose complexity is further compounded by their high rate of comorbidity with other psychiatric disorders, especially depression and anxiety (Mitchell et al., 2014). Given the resultant high morbidity and mortality (Fisher et al., 2001; Petkova et al., 2019), early identification and prompt treatment are crucial. Experts have recently suggested that we have greatly underestimated the prevalence of eating disorders, due to stigma and the tendency to conceal difficulties (Zipfel et al., 2022). Worryingly, presentations of young people with eating disorders to health services increased rapidly during the Coronavirus (COVID‐19) pandemic in both high‐ and low‐income countries (Feinmann, 2021). For example, reports indicate a doubling in admissions for re‐feeding in Australia (Haripersad et al., 2021) and urgent referrals in England during 2020, combined with a smaller increase in nonurgent referrals (NHS England, 2022). While the number of young people seeking treatment has increased, only assessments of population‐based samples can differentiate between an increase in the underlying symptomology or a change in treatment‐seeking behavior, which is important to clarify so that policy and commissioning responses are evidence based.

The Mental Health of Children and Young People in England (MHCYP) survey (Vizard et al., 2018) was commissioned to estimate the prevalence of mental health conditions, including eating disorders. The MHCYP surveys used the Development and Well‐Being Assessment (DAWBA), a multi‐informant standardized diagnostic assessment, to assess mental health among a probability sample of 9117 2‐to‐19‐year‐olds. The DAWBA (Goodman et al., 2000) includes structured and semi‐structured questions within modules that cover most mental health conditions. There are parallel versions for children and young people aged 11 years or more and parents/carers, which can be administered via interview or completed online. A brief questionnaire version is available for teachers. Each module includes “screening items,” which aim to select those reporting any difficulties related to that disorder for further detailed structured questions and semi‐structured probes. Clinical raters assess data from all informants to make a clinical judgment about the likelihood that the child or young person meets diagnostic criteria for that disorder. The DAWBA has been widely used in clinical practice and research (Aebi et al., 2012; Moya et al., 2005).

Given the international rise in presentations to services with eating disorders during the COVID‐19 pandemic (Feinmann, 2021; Zipfel et al., 2022), the follow ups of MHCYP in 2021 and 2022 included the DAWBA eating disorder screening items to allow direct comparison of eating difficulties with 2017. Unfortunately, we lacked time or funding to complete the full DAWBA eating disorder module for those who screened positive, but policymakers and commissioners need to understand how screening positive relates to the prevalence of eating disorders in this population.

The 2021 follow‐up survey report has indicated a doubling in the proportion of 11–16‐year‐olds screening positive on the DAWBA eating disorder screen since 2017 (from 6.7% to 13.0%) and an increase from 44.6% to 58.2% among 17–19‐year‐olds (Williams et al., 2021). It is crucial that we understand how these reports of eating difficulties on the screening items predict an eating disorder diagnosis.

We examined the diagnostic accuracy of the DAWBA screening questions, with the aim of estimating the prevalence of eating disorders from the 2021 follow‐up data. Our objective was to provide empirical context to better assess the extent to which increased clinical demand is being driven by increased population prevalence and to examine the impact of COVID‐19 (Newlove‐Delgado et al., 2021; Williams et al., 2021), by comparing the estimated prevalence to that measured in 2017. Study 1 explored whether the informant (parent or child/young person), the threshold score, or the diagnostic classification influenced the diagnostic accuracy of the DAWBA eating disorders screen and addressed this by using two different false negative rates (described in *Methods*). Study 2 then applied the Positive Predictive Values (PPV) generated from Study 1 to estimate the prevalence of eating disorders in 2021 compared to that in 2017.

We hypothesized that:

1. Parent and child reports combined would yield the highest diagnostic accuracy.
2. The standard DAWBA screen threshold of one positive answer for children/young people would capture more cases than a cut point of two (currently used for parents).
3. Diagnostic accuracy would not differ between the Diagnostic and Statistical Manual of Mental Disorders, 5th edition (DSM‐5) (American Psychiatric Association, 2013) or the International Classification of Diseases, 10th edition (ICD‐10) (World Health Organization, 1992).
4. Estimates of eating disorder prevalence would suggest a significant population increase.

## 2 METHODS

### 2.1 Ethical considerations

The survey was reviewed and approved by the West London & GTAC Research Ethics Committee (16/LO/0155) and the Health Research Authority Confidentiality Advisory Group (16/CAG/0016). The follow‐up surveys were approved by the Office for National Statistics Ethical Committee, and our analysis by the University of Cambridge Psychology Research Ethics Committee (PRE.2020.145).

### 2.2 Study 1: The accuracy of the DAWBA screen in MHCYP 2017

The 2017 MHCYP survey involved a stratified probability sample of 9117 children and young people aged 2–19 years living in England drawn from the NHS Patient Register. More detail about the sampling approach and sample are available in the NHS Digital report (Vizard et al., 2018). Children or young people, and one of their respective parents were invited to complete the DAWBA interview face‐to‐face with trained lay interviewers. For children who were aged 16 and under, parents were interviewed first with permission sought from the parent to interview their child. Children provided assent. Conversely, 17–19‐year‐olds were directly asked for their consent, with permission subsequently sought for their parents to be interviewed. We report results for the 11–16‐year‐olds and the 17–19‐year‐olds separately due to this difference in assessment.

Each module of the DAWBA (including behavioral disorders, anxiety, depression, and neurodevelopmental disorders as well as eating disorders) applies questions to identify those with no problems to proceed directly to the next module, reducing participant burden. Those who screened positive are asked more detailed structured questions that relate directly to the diagnostic criteria, while semi‐structured probes explore the experience of these difficulties (see full DAWBA eating disorders module in Data S1). A small team of expert clinical raters drawn from psychiatry, pediatrics, and public health (including TF and TND) reviewed data from informants to assign diagnoses according to DSM‐5 and ICD‐10 criteria, which slightly differ (see Data S2).

**FIGURE 1:**
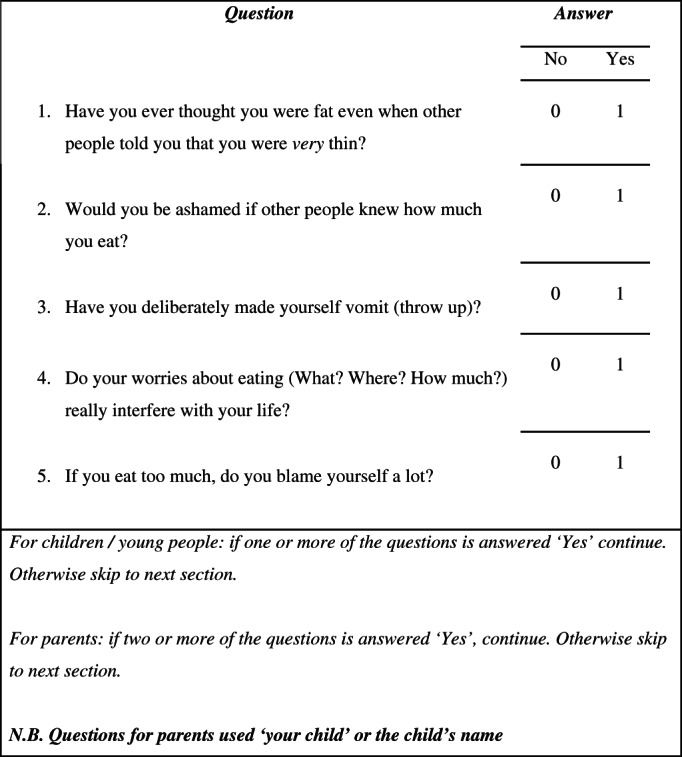
The Development and Well‐Being Assessment eating disorder screening questions

The DAWBA eating disorder module was developed for the 2004 British Child and Adolescent Mental Health Survey (BCAMHS), which involved data from a community sample of 500 young people and their parents (Meltzer et al., 2003), and a sample of 174 Brazilian girls aged 7–17 (48 with eating disorders, 55 clinical controls, and 71 community controls; Moya et al., 2005). This work established that a threshold of two positive answers for parents or one for children/young people, for the five screening questions (see Figure 1), combined with the rest of the eating disorders module, resulted in specificity and sensitivity for a diagnosis of any eating disorder of 94% and 100%, respectively (Moya et al., 2005). The rationale behind the lower threshold for young people is based on the complex, often well‐hidden symptomology of eating disorders (Couturier & Lock, 2006; Vandereycken & van Humbeeck, 2008; Viglione et al., 2006).

### 2.3 Diagnostic accuracy of the eating disorder screening items in the MHCYP 2017

We use the clinically rated multi‐informant DAWBA diagnoses of eating disorders in MHCYP 2017 as the reference standard. We provide diagnostic accuracy measures based on children who were diagnosed with any eating disorder included in the ICD‐10 or DSM‐5. There were very few eating disorders cases as this was an epidemiological sample, so sub‐type analyses may have revealed the identity of the patients.

**FIGURE 2:**
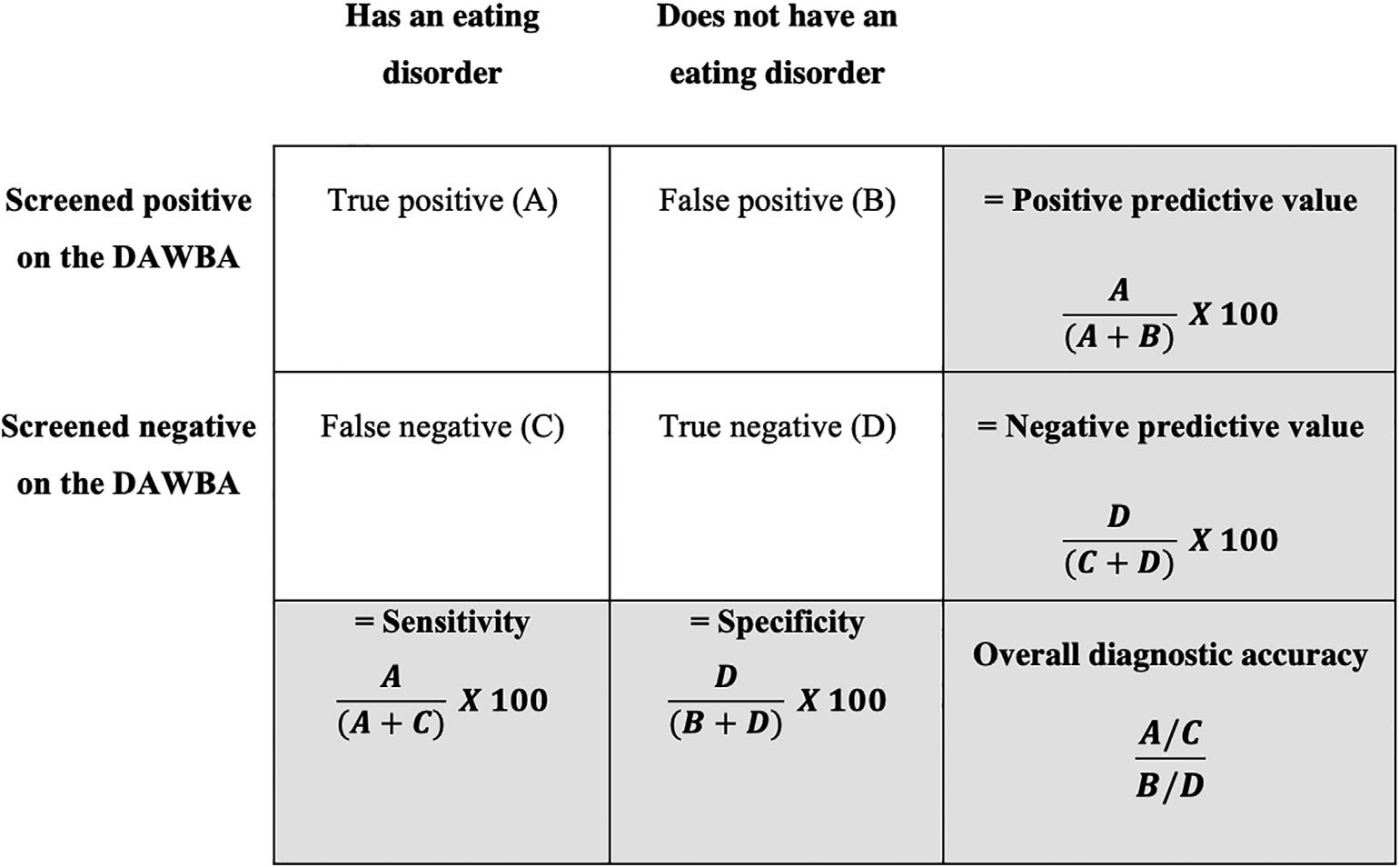
Diagram demonstrating the basis for deriving sensitivity, specificity, and positive and negative predictive values

The index test was whether the informant scored above or below the threshold on the DAWBA screening questions: (i) as it is normally applied or (ii) set at two positive items for both parents and young people. We assessed the diagnostic accuracy of (1) these two different thresholds, (2) ICD‐10 and DSM‐5 criteria, and (3) individual responses versus combined responses from the parent and child or young person (where available). The components of diagnostic accuracy are defined in Figure 2, along with how they are calculated. They include PPV, Negative Predictive Value (NPV), specificity and sensitivity. Sensitivity is the ability of a test to correctly identify patients with a condition (percentage of true positives) while specificity is the ability to correctly identify people without the condition (percentage of true negatives). PPV and NPV are the probabilities that the test correctly identifies an individual as having a specific condition or not following a positive or negative test result, respectively. The overall diagnostic accuracy of the DAWBA screening questions was its ability to detect a condition when it is present and the absence of a condition when it is absent.

The 2017 MHCYP routed all children and parents who screened negative through to the next module, which led to zero false negatives. A study designed to establish test accuracy would collect data on some screen negatives but because this was a survey that was not designed to do that, we applied false negative rates from a previous validation study of the DAWBA screen based on the 2004 BCAMHS (Meltzer et al., 2003). This involved 500 participants from a community‐based sample and 41 participants from a clinical sample who completed the full DAWBA eating disorders module with no skip rules (Meltzer et al., 2003). Applying the screen to these data produced no false negatives in the community sample but failed to detect 1/41 (2.4%) eating disorders in the clinical sample. Therefore, we ran our analysis twice, once assuming zero false negatives (0%) and once assuming a false negative rate of 2.4%.

### 2.4 Study 2: Application of estimated PPVs to the 2021 MHCYP survey findings

**FIGURE 3:**
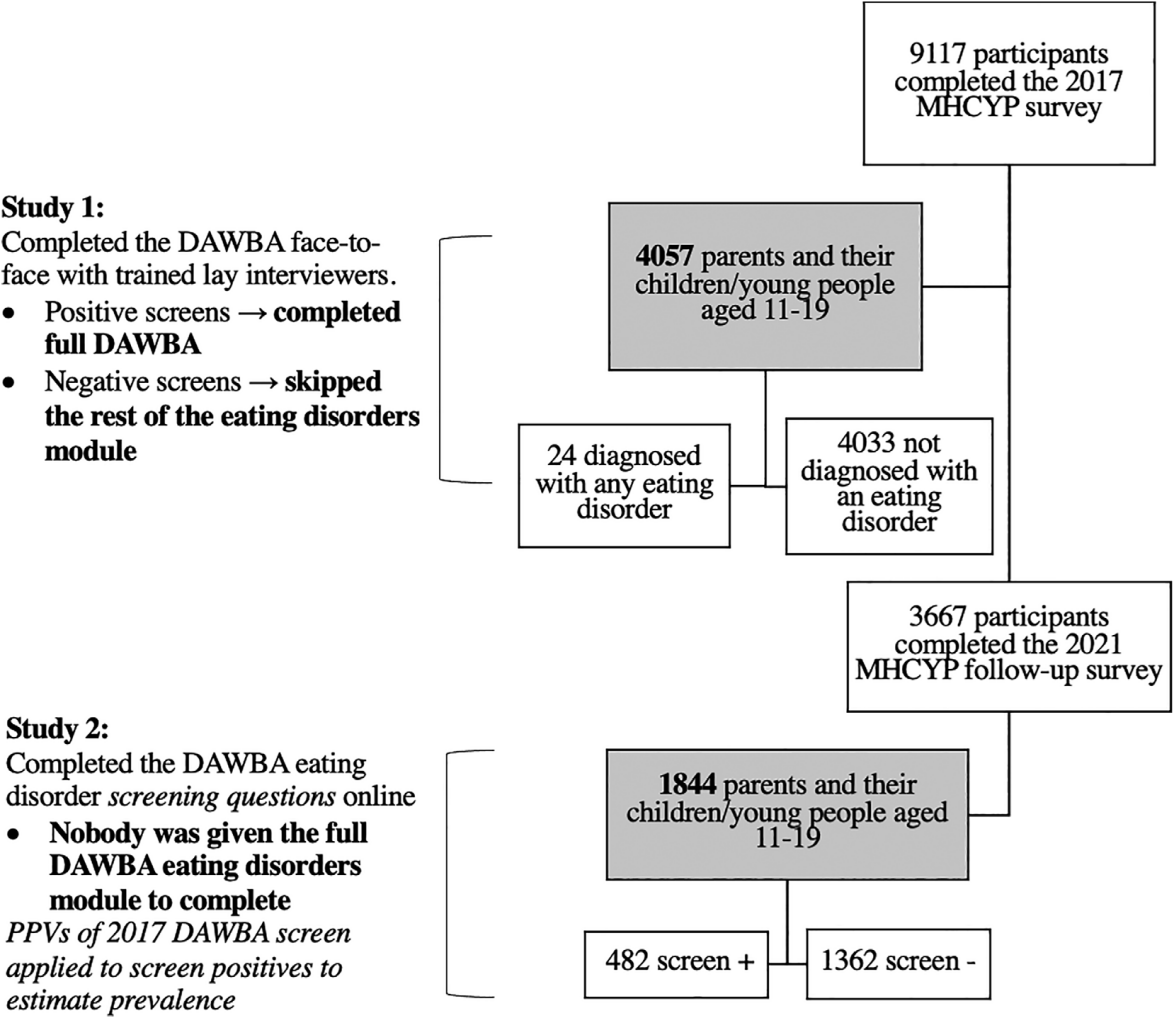
Flow diagram and Development and Well‐Being Assessment administration for each study

All participants who consented to re‐contact in 2017 were invited by mail to complete a brief questionnaire, which included the eating disorders screening questions. As previously, parents reported for their children under the age of 16 and young people aged 11 and over completed their own reports. In 2021, a total of 3667 participants completed the follow‐up survey, which included 1844 participants aged 11–19 (Williams et al., 2021). Figure 3 shows the flow diagrams of participants and methods for both Studies 1 and 2.

Since none of the participants in 2021 could be diagnosed with an eating disorder, we applied the estimated PPVs from Study 1 to those who “screened positive” in this age group in MHYCP 2021 to provide a crude estimate of the proportion of those screening positive in 2021 whom we would expect to meet diagnostic criteria for an eating disorder if they had completed the full DAWBA eating disorders module. We used the PPVs from parent‐reported estimates for 11–16‐year‐olds, and self‐reported estimates for 17–19‐year‐olds, as data for only these groups were available in the published 2021 report (raw data not yet available for analysis) (Williams et al., 2021). PPV confidence intervals were also used to generate a range for these 2021 eating disorder prevalence estimates.

## 3 RESULTS

### 3.1 Study 1: How does the DAWBA eating disorder screen relate to the diagnosis of eating disorders in MHCYP 2017?

#### 3.1.1 Accuracy of the DAWBA screen assuming no false negatives

Table 1 reports the diagnostic accuracy measures for the standard DAWBA screen threshold for children and their parents in 2017, based on the assumption of no false negatives. Test accuracy estimates for the standard thresholds were similar for both ICD‐10 and DSM‐5 diagnoses, with no significant differences (*p* = .2, t(14) = 1.3). The text below, therefore, refers to DSM‐5 diagnoses unless otherwise specified.

**TABLE 1:** Measures of diagnostic accuracy of the standard DAWBA score threshold for an eating disorder diagnosis according to DSM‐5 and ICD‐10 criteria for children aged 11–16 years old, young people aged 17–19 years old and their respective parents (with 95% confidence intervals) in 2017 based on zero false negatives

Informant type	Diagnostic criteria used for clinical rating	Positive predictive value (%)	Negative predictive value (%)	Sensitivity (%)	Specificity (%)	Accuracy (%)
Parents of 11–16‐year‐olds (*n* = 3112)	DSM‐5	4.9 (1.9–7.9)	99.8 (99.6–100.0)	66.7 (42.8–90.5)	93.7 (92.8–94.6)	93.6 (92.7–94.5)
	ICD‐10	4.4 (1.6–7.2)	99.8 (99.6–100.0)	64.3 (39.2–89.4)	93.7 (92.8–94.6)	93.6 (92.7–94.5)
Children aged 11–16 years (*n* = 2597)	DSM‐5	1.0 (.9–1.1)	100.0 (‐)	100.0 (66.4–100.0)	65.7 (63.4–67.5)	65.8 (63.9–67.6)
	ICD‐10	.9 (‐)	100.0 (‐)	100.0 (63.1–100.0)	65.6 (63.8–67.5)	65.7 (63.9–67.6)
Parents and their children 11–16 years combined (*n* = 2591)	DSM‐5	.8 (.7–.8)	100.0 (‐)	100.0 (66.4–100.0)	55.2 (53.3–57.1)	55.4 (53.4–57.3)
	ICD‐10	.7 (.6–.7)	100.0 (‐)	100.0 (63.1–100.0)	55.2 (53.2–57.1)	55.3 (53.4–57.2)
Parents of 17–19‐year‐olds (*n* = 415)	DSM‐5 & ICD‐10^a^	6.3 (4.5–8.6)	100.0 (‐)	100.0 (15.8–100.0)	92.7 (89.8–95.1)	92.8 (89.8–95.1)
Young people aged 17–19 years old (*n* = 935)	DSM‐5 & ICD‐10^a^	1.7 (1.6–1.8)	100.0 (‐)	100.0 (59.0–100.0)	55.3 (52.0–58.5)	55.6 (52.4–58.8)
Parents and their children aged 17–19 years combined (*n* = 413)	DSM‐5 & ICD‐10^a^	.9 (.8–1.0)	100.0 (‐)	100.0 (15.8–100.0)	47.9 (43.0–52.9)	48.2 (43.3–53.1)

^a^ Both diagnostic criteria detected the same cases.

Using the standard threshold, the screening questions were highly *specific* in parents: 93.7 (95% CI: 92.8–94.6) for parents of 11–16‐year‐olds, and 92.7% (95% CI: 89.8–95.1) for parents of 17–19‐year‐olds, and highly *sensitive* in children and young people at 100%.

Table 2 shows the diagnostic accuracy estimates for parents and their children when a threshold of two positive answers on the screen was applied to both informants. The higher threshold for children and young people increased overall accuracy, but at the expense of increasing the number of false negatives—80% of children/young people who met diagnostic criteria screened positive when a threshold of two or more was applied to both informants. Sensitivity was highest when parent and child answers were combined (Table 2).

**TABLE 2:** Measures of diagnostic accuracy of the DAWBA score threshold of 2+ applied to children aged 11–16 years, young people aged 17–19 years and combined with their respective parents (where both provided responses) for an eating disorder diagnosis according to DSM‐5 and ICD‐10 criteria (with 95% confidence intervals) in 2017 based on zero false negatives

Informant type	Diagnostic criteria used for clinical rating	Positive predictive value (%)	Negative predictive value (%)	Sensitivity (%)	Specificity (%)	Accuracy (%)
Children aged 11–16 years (*n* = 2597)	DSM‐5	1.9 (1.3–2.6)	99.9 (99.7–100.0)	77.8 (40.0–97.2)	85.7 (84.3–87.0)	85.6 (84.2–87.0)
	ICD‐10	1.6 (1.1–2.4)	99.9 (99.7–100.0)	75.0 (34.9–96.8)	85.6 (84.2–87.0)	85.6 (84.2–86.9)
Parents and their children 11–16 years combined (*n* = 2591)	DSM‐5	2.4 (1.9–3.1)	100.0 (99.7–100.0)	88.9 (51.8–99.7)	87.3 (86.0–88.6)	87.3 (86.0–88.6)
	ICD‐10	2.1 (1.6–2.8)	100.0 (99.7–100.0)	87.5 (47.4–99.7)	87.3 (86.0–88.6)	87.3 (86.0–88.6)
Young people aged 17–19 years (*n* = 935)	DSM‐5 & ICD‐10^a^	3.8 (3.3–4.3)	100.0 (‐)	100.0 (59.0–100.0)	80.7 (78.0–83.2)	80.8 (78.1–83.3)
Parents and their children aged 17–19 years combined (*n* = 413)	DSM‐5 & ICD‐10^a^	2.5 (2.1–3.1)	100.0 (‐)	100.0 (15.8–100.0)	81.3 (77.2–84.9)	81.4 (77.3–85.0)

^a^ Both diagnostic criteria detected the same cases.

#### 3.1.2 Accuracy of the DAWBA screen assuming a false positive rate of 2.4%

Table 3 depicts the diagnostic accuracy measures for the use of the standard DAWBA screen threshold for children and their parents in 2017 when the false negative rate of 2.4% was applied.

**TABLE 3:** Measures of diagnostic accuracy of the standard DAWBA score threshold and an eating disorder diagnosis according to DSM‐5 and ICD‐10 criteria for children aged 11–16 years old, young people aged 17–19 years old and their respective parents (with 95% confidence intervals) in 2017 based on applied false negative rate from clinical sample (2.4%)

Informant type	Diagnostic criteria used for clinical rating	Positive predictive value (%)	Negative predictive value (%)	Sensitivity (%)	Specificity (%)	Accuracy (%)
Parents of 11–16‐year‐olds (*n* = 3112)	DSM‐5	4.9 (1.9–7.9	97.5 (96.8–98.0)	11.8 (4.9–18.6)	93.6 (92.7–94.5)	91.4 (90.3–92.3)
	ICD‐10	4.4 (1.6–7.2)	97.4 (96.8–98.0)	10.7 (4.1–17.3)	93.6 (92.7–94.5)	91.3 (90.3–92.3)
Children aged 11–16 years (*n* = 2597)	DSM‐5	1.0 (.6–1.8)	97.6 (97.3–97.9)	18.0 (8.6–31.4)	65.1 (63.2–67.0)	64.2 (62.3–66.0)
	ICD‐10	.9 (.5–2.5)	97.6 (97.3–97.9)	16.3 (7.3–29.7)	65.1 (63.2–66.9)	64.2 (62.3–66.0)
Parents and their children 11–16 years combined (*n* = 2591)	DSM‐5	.8 (.4–1.4)	97.6 (97.2–98.0)	20.9 (10.0–36.0)	54.6 (52.6–56.5)	54.0 (52.1–56.0)
	ICD‐10	.7 (.4–1.3)	97.6 (97.2–97.9)	19.1 (8.6–34.1)	54.6 (52.6–56.5)	54.0 (52.1–55.9)
Parents of 17–19‐year‐olds (*n* = 415)	DSM‐5 & ICD‐10^a^	6.3 (1.8–19.7)	97.7 (96.9–98.2)	18.2 (2.3–51.8)	92.6 (89.6–94.9)	90.6 (87.4–93.2)
Young people aged 17–19 years old (*n* = 935)	DSM‐5 & ICD‐10^a^	1.7 (.9–3.0)	97.7 (96.7–98.3)	36.8 (16.3–61.6)	54.7 (51.4–58.0)	54.3 (51.1–57.6)
Parents and their children aged 17–19 years combined (*n* = 413)	DSM‐5 & ICD‐10^a^	.9 (.3–2.9)	97.5 (96.0–98.4)	28.6 (3.7–71.0)	47.3 (42.4–52.3)	47.0 (42.1–51.9)

^a^ Both diagnostic criteria detected the same cases.

Compared with Table 1, NPV was high for all informants but particularly high for parents, regardless of the false negative rate or threshold. PPV was extremely low for all informants under all conditions, as would be expected given the relatively low prevalence of eating disorders among this age group at population level and the design of the DAWBA to screen with extensive additional assessment of screen positives.

Parental report was highly specific for both age groups and both diagnostic classifications, but sensitivity estimates were imprecise and lower for 11–16‐year‐olds than 17–19‐year‐olds. Applying the false negative rate greatly reduced the sensitivity of the screening questions across all informants. Sensitivity was higher when parent and child answers were combined, and even higher when the threshold for children was two or more compared to parents as single informants (see Table 4 below). The higher threshold for young people increased overall accuracy, but at the expense of increasing false negatives.

**TABLE 4:** Measures of diagnostic accuracy of the DAWBA score threshold of 2+ applied to children aged 11–16 years, young people aged 17–19 years and combined with their respective parents (where both provided responses) for an eating disorder diagnosis according to DSM‐5 and ICD‐10 criteria (with 95% confidence intervals) in 2017 based on applied false negative rate from clinical sample (2.4%)

Informant type	Diagnostic criteria used for clinical rating	Positive predictive value (%)	Negative predictive value (%)	Sensitivity (%)	Specificity (%)	Accuracy (%)
Children aged 11–16 years (*n* = 2597)	DSM‐5	1.9 (.9–3.7)	97.5 (97.3–97.7)	11.3 (4.7–21.9)	85.4 (83.9–86.7)	83.6 (82.1–85.0)
	ICD‐10	1.6 (.7–3.4)	97.5 (97.3–97.7)	9.8 (3.7–20.2)	85.3 (83.9–86.7)	83.6 (82.1–85.0)
Parents and their children 11–16 years combined (*n* = 2591)	DSM‐5	2.4 (1.3–4.5)	97.6 (97.3–97.8)	12.7 (5.7–23.5)	87.1 (85.7–88.4)	85.3 (83.8–86.6)
	ICD‐10	2.1 (1.0–4.1)	97.6 (97.3–97.8)	11.3 (4.7–21.9)	87.0 (85.7–88.3)	85.2 (83.8–86.6)
Young people aged 17–19 years old (*n* = 935)	DSM‐5 & ICD‐10^a^	3.8 (2.0–6.9)	97.6 (96.9–98.1)	28.0 (12.1–49.4)	80.3 (77.5–82.8)	78.9 (76.1–81.4)
Parents and their children aged 17–19 years combined (*n* = 413)	DSM‐5 & ICD‐10^a^	2.5 (.7–8.4)	97.6 (96.8–98.2)	20.0 (2.5–55.6)	80.9 (76.7–84.6)	79.4 (75.2–83.2)

^a^ Both diagnostic criteria detected the same cases.

### 3.2 Study 2: Estimating prevalence by applying the PPVs to those who screened positive in the 2021 MHCYP follow‐up survey

Table 5 displays crude eating disorder prevalence estimates for 11–19‐year‐olds in 2021 generated by applying our estimated PPVs and 95% confidence intervals to the proportion of children and young people screening positive in the 2021 MHCYP surveys. The overall estimates show little difference between 2017 and 2021. However, the range of prevalence estimates for young men aged 17–19 was between .4% and 1.2% compared to a prevalence estimate of below .1 in 2017, while for younger boys, the lower limit of the range in 2021 equates to the 2017 prevalence. Similarly, the point prevalence estimate for young women represents a 20% increase in prevalence between 2017 and 2021, but the range of estimates varies from a 30% decrease to nearly doubling.

**TABLE 5:** Application of estimated PPVs from 2017 to children and young people screening positive in the 2021 MHCYP

	Proportion screening positive in 2017 MHCYP survey (%)	DSM‐5/ICD‐10 eating disorder prevalence, 2017 (%)	Proportion screening positive in 2021 MHCYP survey (%)	Estimates of DSM‐5/ICD‐10 eating disorder prevalence using PPV estimates, 2021 (%)		
		Estimate		Estimate	Lower limit (from 95% CI for PPV)	Upper limit (from 95% CI for PPV)
11–16‐year‐olds (parent report)						
All	6.5	.6	13.0	.6	.2	1.0
Girls	8.4	1.0	17.8	.9	.3	1.4
Boys	5.1	.2	8.4	.4	.2	.7
17–19‐year‐olds (self‐report)						
All	45.1	.8^a^	58.2	1.0	.5	1.7
Girls	60.5	1.6^a^	76.4	1.3	.7	2.3
Boys	29.6	.0^a^	41.0	.7	.4	1.2

^a^ Prevalence figures for 2017 draw on a mix of 17–19‐year‐olds and their parents' reports while in 2021, 17–19‐year‐olds self‐reported only.

## 4 DISCUSSION

We aimed to provide empirical context to explain the increased clinical presentations of young people with eating disorders through exploring the diagnostic accuracy of the DAWBA screen and applying the results to the 2021 reports of eating difficulties. MHCYP needed to apply the same measure in 2017 and 2021 in order to make a direct comparison so we were constrained by the choice of measure.

The DAWBA screening questions were most strong at ruling *out* an eating disorder in children and young people, which is what they were designed to do (Goodman et al., 2000). The DAWBA uses skip rules to balance participant burden against diagnostic accuracy and aims to select out those with no problems in the knowledge that more detailed assessment will support the differentiation of clinical from subclinical disorders. Overall, parent reports were the most diagnostically accurate and specific, which suggests that clinicians can mostly be reassured by a lack of parental concern regarding their child's eating, consistent with the current literature (Ford et al., 2005).

The assessment of eating disorders is complicated as clinical detection relies heavily upon a patient's willingness and ability to share information about their eating behaviors. The denial of symptoms is common, particularly among people with anorexia nervosa (Couturier & Lock, 2006). Although some behaviors are observable, they may not be reported by family, peers and clinicians unless directly enquired about. Furthermore, some of the symptoms required for an eating disorder diagnosis to be made, according to criteria from the DSM‐5 or ICD‐10, are complex and can be difficult to operationalize in a short screening questionnaire. Existing screening items are either limited to certain age ranges and often exclude younger adolescents to focus on teenagers and adults (e.g., the SCOFF questions [Morgan et al., 1999]), include eight or more items (e.g., Children's Eating Disorder Examination‐Questionnaire, ChEDE‐Q8 [Kliem et al., 2017]), or focus on particular behaviors (e.g., Adolescent Binge Eating Questionnaire, ADO‐BED [Chamay‐Weber et al., 2017]). We could benefit from brief general screen that could be used to assess population prevalence as well as for nonspecialists to identify young people who may be struggling with their eating and need further clinical assessment (Zipfel et al., 2022).

Denial and minimization of illness is common among individuals with eating disorders (Starzomska & Tadeusiewicz, 2016; Viglione et al., 2006). Our findings indicate that raising the threshold for young people reduces false positives (fewer unnecessary additional assessments) at the expense of false negatives (more undetected cases). How much this matters depends on the reason for using the screen and opportunities for additional assessment. For example, when positive screens automatically lead to detailed assessment, such as in the 2017 MHCYP survey, minimizing false negatives will ensure more accurate prevalence estimates. In contrast, minimizing false negatives in clinical assessments by school nurses or general practitioners is essential, especially given the low PPV and potential to raise anxiety and swamp clinical services. Eating disorders are highly persistent with a proven mortality and given that their prevalence is probably greatly underestimated, the lower threshold for young people is recommended, with additional assessment by the screening practitioner before referral to specialist services.

Combining parental and child reports increased the sensitivity of the DAWBA screen, but reduced specificity and overall accuracy. Discrepancies between young people and adult informants on mental health symptoms are one of the most robust findings in child and adolescent psychiatry (Collishaw et al., 2009). Information from young people is particularly helpful for detecting concealed or internally experienced difficulties such as self‐harm, depression, and anxiety, while information from teachers and parents is more useful with reference to neurodevelopmental and behavioral problems (Aebi et al., 2017; Ford et al., 2005; Kuhn et al., 2017). Both denial of symptoms and lack of insight may also contribute to the lower specificity and moderate sensitivity of the screening questions for young people in our analysis (Keski‐Rahkonen et al., 2006; Vandereycken & van Humbeeck, 2008). The strongest predictor of disordered eating behaviors later in adolescence is the degree of disordered eating already present in early adolescence (Attie & Brooks‐Gunn, 1989; Wichstrøm, 2000) which suggests that disordered eating, once present, tends not to resolve spontaneously and intensifies over time. Parent or carer reports can therefore still be important and informative during emerging adulthood.

We urge caution when interpreting of the prevalence estimates, which are based on a crude analysis using published proportions rather than raw data. Our 2017 estimates are also inevitably influenced by sampling strategies, age, and the screening tool itself. Nevertheless, we attempted to estimate prevalence for a particular age group in comparison to a particular population‐based sample using a particular measure. Both MHCYP surveys report a relatively low prevalence of eating disorders in young people compared to some current literature using other tools (Mitchison et al., 2020; Nagl et al., 2016; Silen et al., 2020). Yet, the prevalence and incidence patterns of eating disorders remain disputed, mainly because of different assessment methods and sampling strategies, as well as recent changes in diagnostic criteria. Swanson et al. reports similar lifetime prevalence of eating disorder to what we found in our study, among boys and girls aged 13–18 using the Composite International Diagnostic Interview (Swanson et al., 2011). In contrast, the German KiGGS‐BELLA study used the SCOFF questionnaire to examine disordered eating behavior in a representative sample of 1895 children and adolescents aged 11–17 (Ravens‐Sieberer et al., 2008) and found that overall, 29% of the girls and 14% of the boys reported disordered eating behavior indicating that, among adolescents, subthreshold eating disorders might be considerably more frequent compared to threshold eating disorders.

The DAWBA eating disorder module was designed two decades ago and primarily focused on anorexia and bulimia nervosa but was updated to include DSM‐5 prior to the 2017 survey. The DAWBA tends to generate fewer, more severe cases when compared to other standardized diagnostic instruments, which is why it is recommended for prevalence surveys (Angold et al., 2012).

Despite only tentative evidence of increased prevalence in eating disorder, the proportion of children and young people who “screened positive” on the DAWBA screening questions rose significantly between 2017 and 2021, which is still worrying (Williams et al., 2021). Children and young people with sub‐clinical eating difficulties may still experience impairment and benefit from identification and support. Such symptoms have been strongly associated with other mental health difficulties, including depression, anxiety, substance misuse, and personality disorders (Godart et al., 2007; Hudson et al., 2007; Swanson et al., 2011). It will be important to study the mental health, impairment, and service access trajectory of these children and young people over time to understand better how support and improve their mental health, once the data are available.

Clinical raters could use evidence from other sections of the DAWBA (e.g., body dysmorphia, depression) as well as qualitative data to assign diagnoses if clinical impairment was evident, even if DSM‐5 and ICD‐10 diagnostic criteria were not met, with only one additional eating disorder emerging in this fashion. Thus, despite the limitations of the DAWBA screen, the very high NPV gives us confidence that those screening negative in the 2017 and 2021 MHCYP surveys are unlikely to have an eating disorder. The 2021 follow‐up had to be brief and online, but our work supports the need for inclusion of the full DAWBA in a future baseline population survey with a refreshed and representative sample. Repeating the DAWBA in a larger sample would also provide data on trends in eating disorders, allowing the longer‐term impacts of COVID‐19 on eating behaviors to be better examined.

This is the first study to examine the diagnostic accuracy of the DAWBA screening questions in parents and young people, and the only population‐based study to estimate population prevalence of eating disorders before and after the pandemic. It benefits from a large, representative population sample. Nevertheless, the data were not originally collected with the intention of conducting a diagnostic accuracy study, and the present findings must be interpreted with limitations in mind. The full DAWBA was not applied to participants who screened negative in 2017, which deprived Study 1 of a true false‐negative rate, necessitating assumptions based on a smaller pilot sample. The “true” false negative rate in the general population of children and young people, however, is likely to fall between our two estimates (0%–2.4%) using this tool.

Further research should analyze the results from the full DAWBA on a large sample of participants who screen negative to ensure more precise estimates. Sensitivity analyses with different assumptions could bolster these results, but the raw data are yet to be made available from NHS Digital.

## 5 CONCLUSION

The DAWBA eating disorder screen shows strong NPV and, particularly when used with parents or carers, could be highly useful at ruling out (if negative) and also ruling in (if positive) an eating disorder in clinical practice. While parental report alone provides the highest diagnostic accuracy, our findings also highlight the importance of speaking to both parents and children during assessment and using clinical judgment to balance the evidence when their accounts conflict. Finally, we found tentative evidence of a modest increase in eating disorders at population level.

## AUTHOR CONTRIBUTIONS

**Tamsin Newlove‐Delgado:** Resources; writing – original draft; writing – review and editing. **Sally McManus:** Data curation; project administration. **Frances Mathews:** Writing – review and editing. **Suzanne Hill:** Data curation; project administration. **Katharine Sadler:** Data curation; methodology. **Tamsin J Ford:** Conceptualization; funding acquisition; supervision; writing – original draft; writing – review and editing.

## FUNDING INFORMATION

The original survey was commissioned by the Department of Health for England and delivered by a consortium including the National Centre of Social Research (NatCen), Office for National Statistics (ONS), and the universities of Cambridge and Exeter. The UKRI funded the wave 2 follow up and the current analysis: *Tracking the impact of COVID‐19 on national longitudinal probability sample MR/V027751/1*. All research at the Department of Psychiatry in the University of Cambridge was supported by the NIHR Cambridge Biomedical Research Centre (BRC‐1215‐20014) and NIHR Applied Research Centre. Tamsin Newlove Delgado was supported by an NIHR Advanced Fellowship (NIHR 300056). The views expressed are those of the author(s) and not necessarily those of the NIHR or the Department of Health and Social Care.

## CONFLICT OF INTEREST

The authors declare no conflict of interest.

## Supporting information


**Data S1** The full DAWBA eating disorders module,

**Data S2** DSM‐5 and ICD‐10 criteria for eating disorders

## Data Availability

The data that support the findings of this study are available from the UK Data Service via NatCen. Restrictions apply to the availability of these data, which were used under license for this study. Data are available from https://doi.org/10.5255/UKDA-SN-8467-2 with the permission of NHS Digital.
