# Supplementary material for: How does the increase in eating difficulties according to the Development and Well‐Being Assessment screening items relate to the population prevalence of eating disorders? An analysis of the 2017 Mental Health in Children and Young People survey
Source: Int J Eat Disord. 2022 Oct 20;55(12):1777–87. doi: 10.1002/eat.23833 (PMC10092017; doi:10.1002/eat.23833)

**SUPPLEMENTARY MATERIAL**

**SM1– the full DAWBA eating disorders module,** [**https://dawba.info/py/dawbainfo/b1list.py?language=English**](https://dawba.info/py/dawbainfo/b1list.py?language=English)


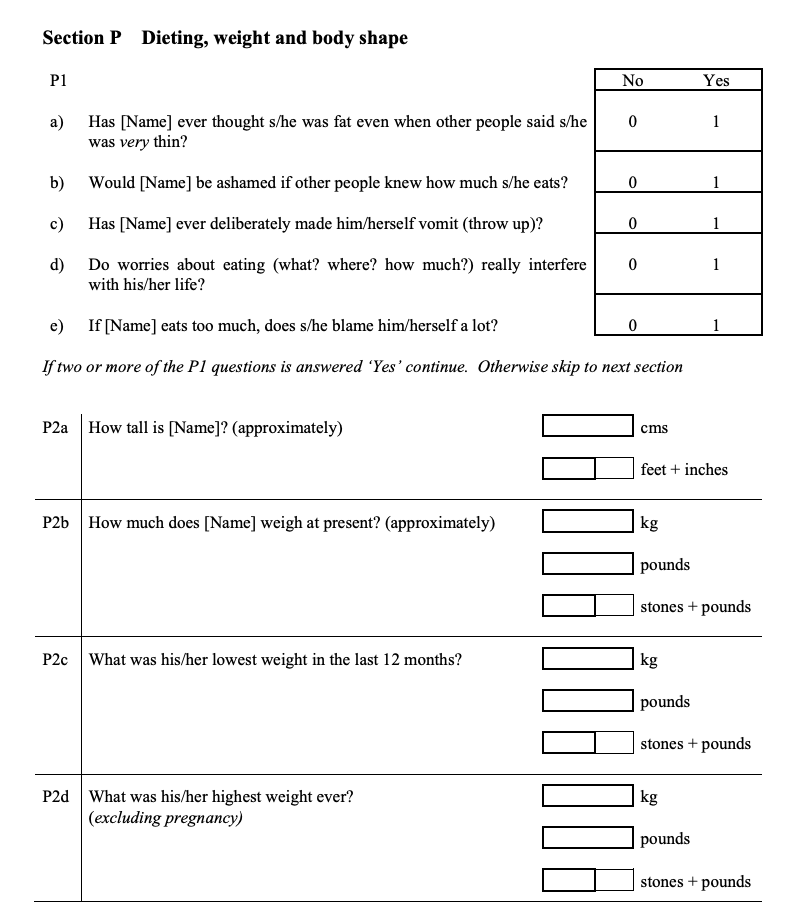


***The 5 screening questions***


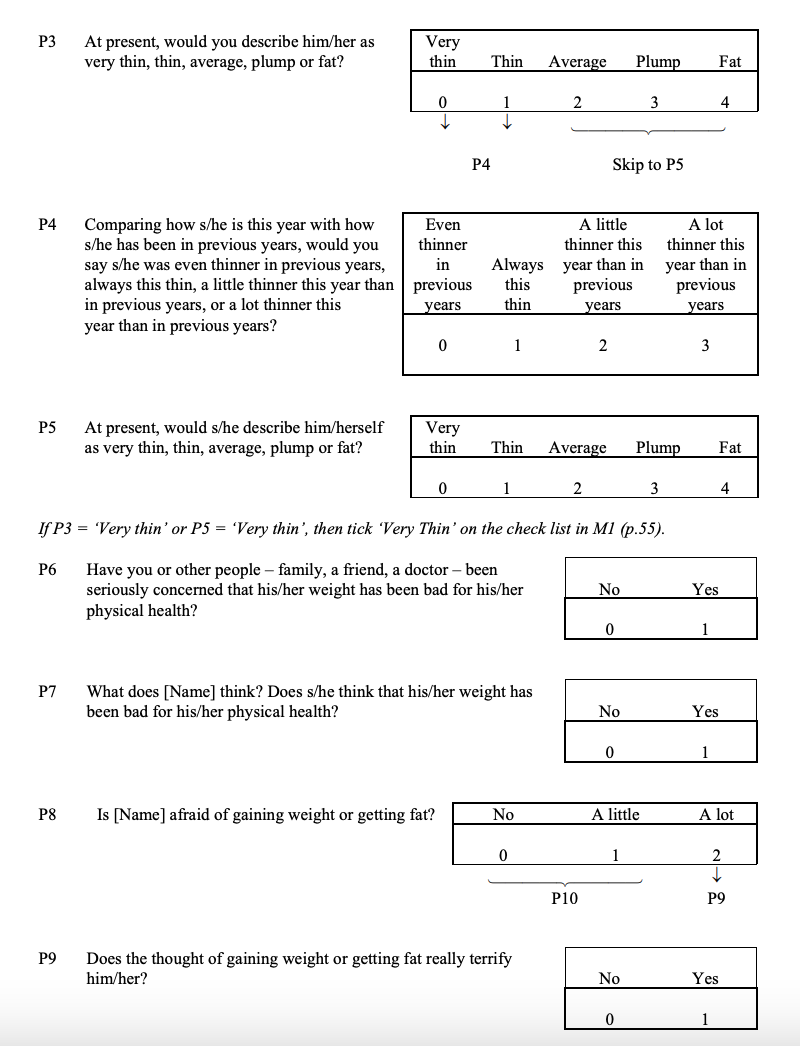


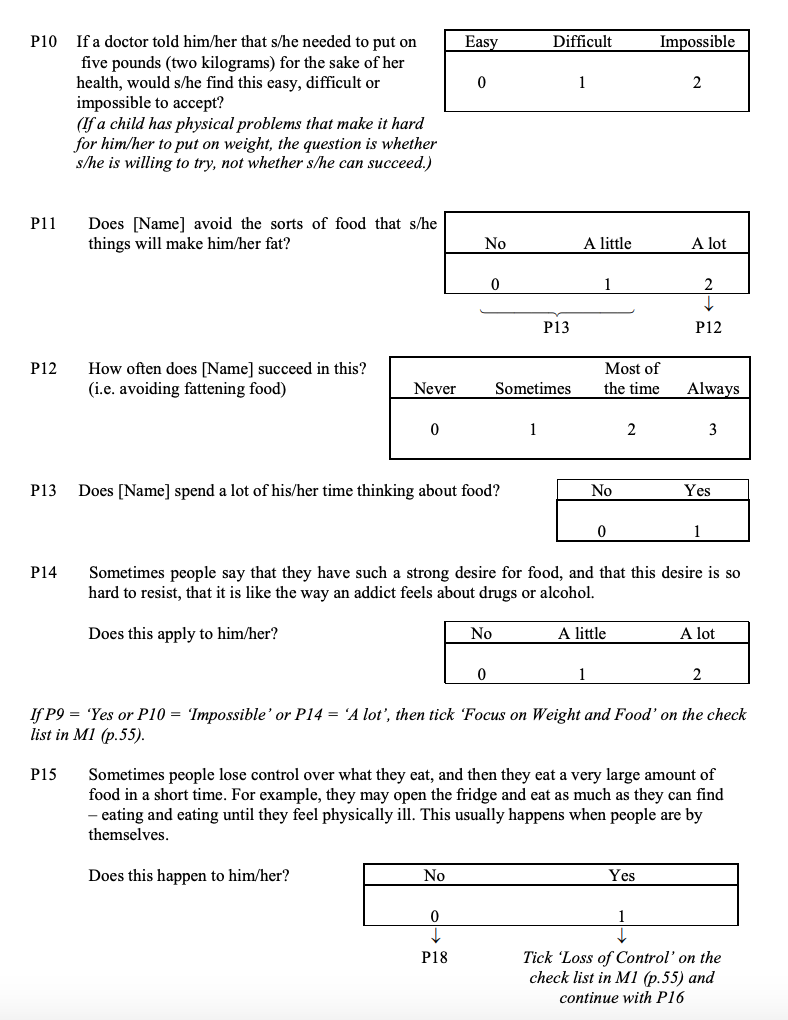


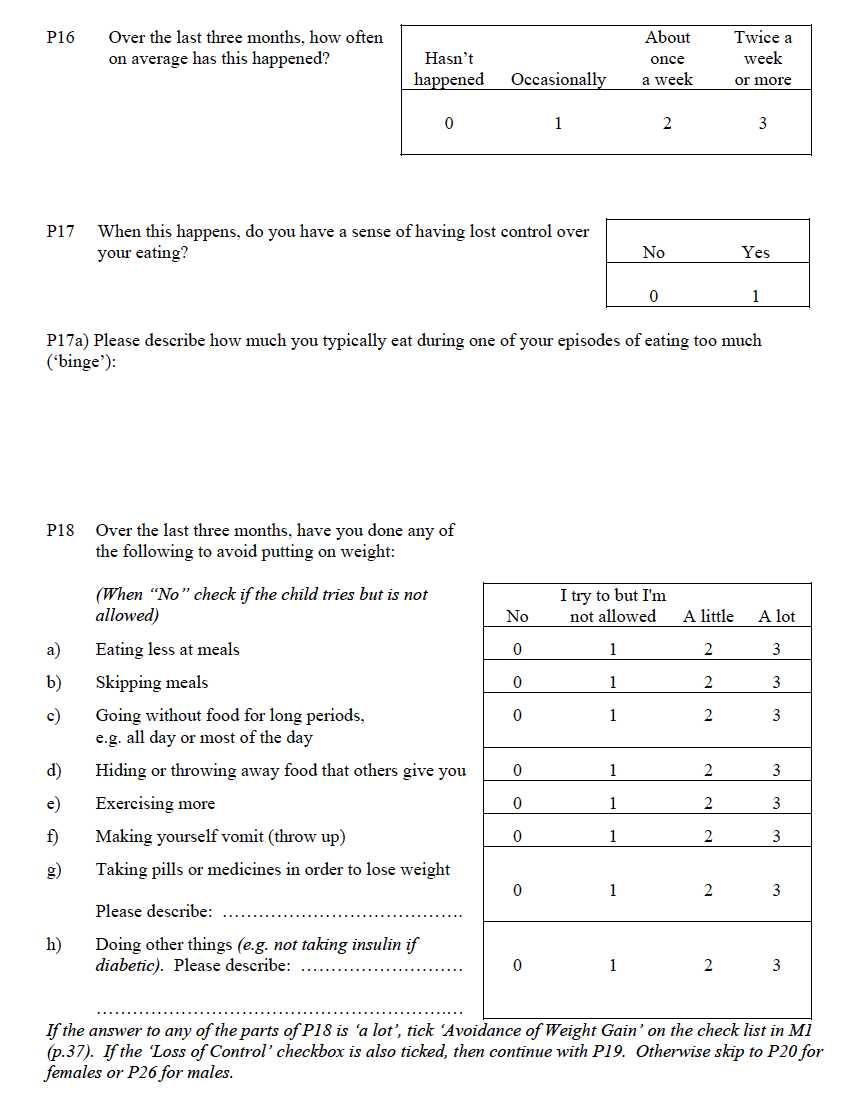


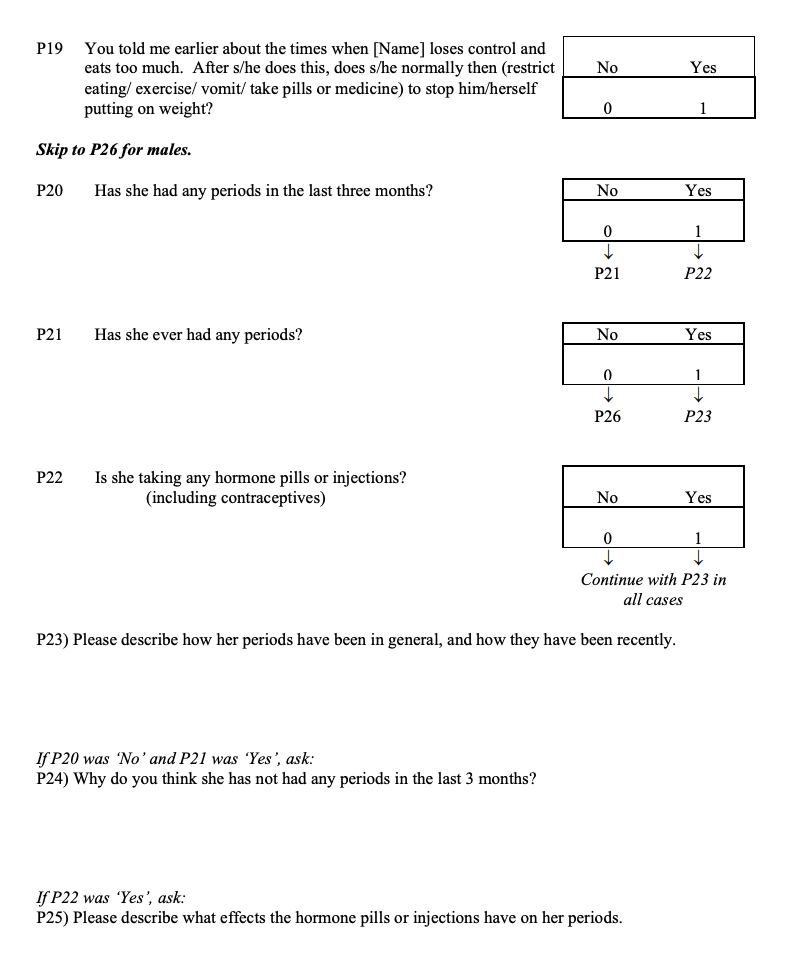

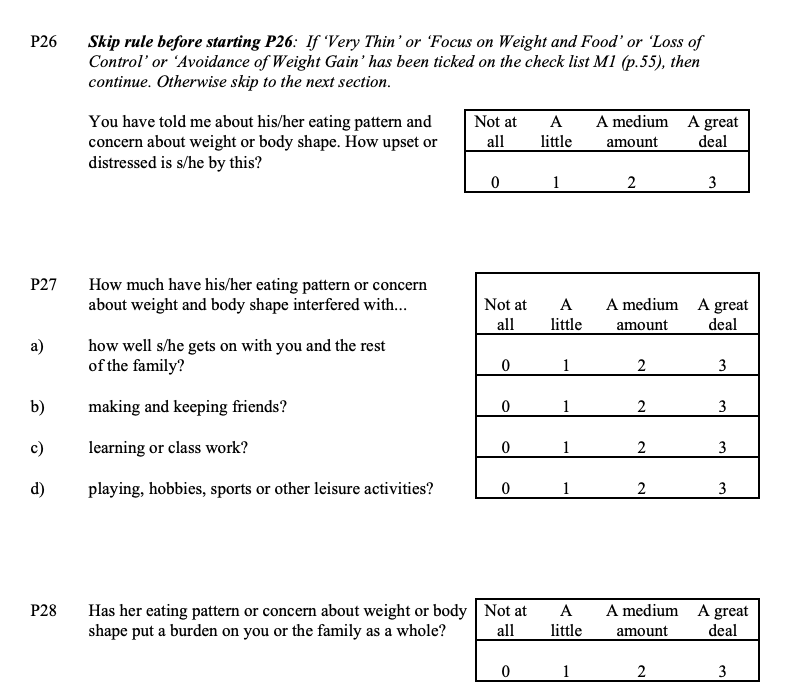

Supplement: Supplementary file 1 — Data S1 The full DAWBA eating disorders module, [file EAT-55-1777-s001.docx]
