# Supplementary material for: How does the increase in eating difficulties according to the Development and Well‐Being Assessment screening items relate to the population prevalence of eating disorders? An analysis of the 2017 Mental Health in Children and Young People survey
Source: Int J Eat Disord. 2022 Oct 20;55(12):1777–87. doi: 10.1002/eat.23833 (PMC10092017; doi:10.1002/eat.23833)
Supplement: Supplementary file 2 — Data S2 DSM‐5 and ICD‐10 criteria for eating disorders [file EAT-55-1777-s002.docx]

**SUPPLEMENTARY MATERIAL**

**SM2: DSM-5 and ICD-10 criteria for eating disorders**

| **Eating disorder** | **DSM-5** | **ICD-10** |
| --- | --- | --- |
| Anorexia nervosa | A. Restriction of energy intake relative to requirements, leading to a significant low body weight in the context of the age, sex, developmental trajectory, and physical health (less than minimally normal/expected).  B. Intense fear of gaining weight or becoming fat or persistent behaviour that interferes with weight gain.  C. Disturbed by one’s body weight or shape, self-worth influenced by body weight or shape, or persistent lack of recognition of seriousness of low bodyweight. | F50.0 Anorexia nervosa  A. There is weight loss or, in children, a lack of weight gain, leading to a body weight at least 15% below the normal or expected weight for age and height.  B. The weight loss is self-induced by avoidance of "fattening foods",  C. There is self-perception of being too fat, with an intrusive dread of fatness, which leads to a self-imposed low weight threshold.  D. A widespread endocrine disorder involving the hypothalamic-pituitary-gonadal axis is manifest in women as amenorrhoea and in men as a loss of sexual interest and potency. (An apparent exception is the persistence of vaginal bleeds in anorexic women who are on replacement hormonal therapy, most commonly taken as a contraceptive pill.)  E. The disorder does not meet criteria A and B for bulimia nervosa (F50.2). |
| Bulimia nervosa | A. Recurrent episodes of binge eating, as characterized by both:  1. Eating, within any 2-hour period, an amount of food that is definitively larger than what most individuals would eat in a similar period of time under similar circumstances.  2. A feeling that one cannot stop eating or control what or how much one is eating.  B. Recurrent inappropriate compensatory behaviours in order to prevent weight gain such as self-induced vomiting; misuse of laxatives, diuretics, or other medications; fasting or excessive exercise.  C. The binge eating and inappropriate compensatory behaviours occur, on average, at least once a week for 3 months  D. Self-evaluation is unjustifiability influenced by body shape and weight.  E. The disturbance does not occur exclusively during episodes of anorexia nervosa. | F50.2 Bulimia nervosa  A. There are recurrent episodes of overeating (at least twice a week over a period of 3 months) in which large amounts of food are consumed in short periods of time.  B. There is persistent preoccupation with eating, and a strong desire or a sense of compulsion to eat (craving).  C. The patient attempts to counteract the "fattening" effects of food by one or more of the following:  a. self-induced vomiting;  b. self-induced purging;  c. alternating periods of starvation;  d. use of drugs such as appetite suppressants, thyroid preparations, or diuretics; when bulimia occurs in diabetic patients, they may choose to neglect their insulin treatment.  D. There is self-perception of being too fat, with an intrusive dread of fatness (usually leading to underweight). |
| ARFID | A. An eating or feeding disturbance (e.g., apparent lack of interest in eating or food; avoidant based on the sensory characteristics of food; concern about aversive consequences of eating) as manifested by persistent failure to meet appropriate nutritional and/or energy needs associated with one (or more) of the following:  1. Significant weight loss (or failure to achieve expected weight gain or faltering growth in children).  2. Significant nutritional deficiency.  3. Dependence on enteral feeding or oral nutritional supplements.  4. Marked interference with psychosocial functioning.  B. The disturbance is not better explained by lack of available food or by an associated culturally sanctioned practice.  C. The eating disturbance does not occur exclusively during the course of anorexia nervosa or bulimia nervosa, and there is no evidence of a disturbance in the way in which one’s body weight or shape is experienced.  D. The eating disturbance is not attributable to a concurrent medical condition or not better explained by another mental disorder. When the eating disturbance occurs in the context of another mental disorder, the severity of the eating disturbance exceeds that routinely associated with the condition or disorder and warrants additional clinical attention. | N/A |
| Binge eating disorder | A. Recurrent episodes of binge eating. An episode of binge eating is characterized by both of the following:  1. Eating, in a discrete period of time (e.g., within any 2-hour period), an amount of food that is definitely larger than most people would eat in a similar period of time under similar circumstances  2. A sense of lack of control overeating during the episode (e.g., a feeling that one cannot stop eating or control what or how much one is eating)  B. The binge-eating episodes are associated with three (or more) of the following:  1. Eating much more rapidly than normal  2. Eating until feeling uncomfortably full  3. Eating large amounts of food when not feeling physically hungry  4. Eating alone because of feeling embarrassed by how much one is eating  5. Feeling disgusted with oneself, depressed, or very guilty after overeating  C. Marked distress regarding binge eating is present.  D. The binge eating occurs, on average, at least 2 days a week for 6 months. Note: The method of determining frequency differs from that used for bulimia nervosa; future research should address whether the preferred method of setting a frequency threshold is counting the number of days on which binges occur or counting the number of episodes of binge eating.  E. The binge eating is not associated with the regular use of inappropriate compensatory behaviours (e.g., purging, fasting, excessive exercise) and does not occur exclusively during the course of anorexia nervosa or bulimia nervosa. | N/A |
| Other specified feeding or eating disorder | Symptoms that are characteristic of a feeing or eating disorder that cause clinical distress or impairment in social, occupational, or other important areas of functioning predominate.  However, DO NOT meet the full criteria for any of the disorders in the feeding and eating disorders diagnostic class.  This category can also be used in situations to communicate the specific reason the presentation does not meet the criteria for a specific eating disorder. E.g., “other specified feeding or eating disorder” followed by the specific reason e.g., “bulimia nervosa- low frequency”.  Examples:  1. Atypical Anorexia Nervosa: all of the criteria for anorexia nervosa are met, except that despite significant weight loss, the individual’s weight is within or above the normal range.  2. Bulimia Nervosa (of low frequency and/or limited duration): all of the criteria for bulimia nervosa are met, except that the binge eating, and inappropriate compensatory behaviours occur, on average, less than once a week and/ or for less than 3 months.  3. Binge-eating disorder (of low frequency and/or limited duration): all of the criteria for binge-eating disorder are met, except that the binge occurs, on average, less than once a week and/ or for less than 3 months.  4. Purging disorder: recurrent purging behaviour to influence weight or shape (e.g., self-induced vomiting; misuse of laxatives, diuretics, or other medications) in the absence of binge eating.  5. Night eating syndrome: Recurrent episodes of night eating, as manifested by eating after awakening from sleep or by excessive food consumption after the evening meal. There is awareness of recall of the eating. The night eating is not better explained by external influences such as changes in the individual’s sleep wake cycle or by local social norms. The night eating causes significant distress and/or impairment in functioning. The disordered pattern of eating is not better explained by binge-eating disorder and or another mental disorder, including substance use, and is not attributable to another medical disorder or to an effect of medication. | Includes: pica of nonorganic origin in adults, binge eating, psychogenic loss of appetite, nocturnal sleep-related eating disorder |
| Unspecified feeding or eating disorder | Symptoms that are characteristic of a feeding and eating disorder & cause clinically significant distress or impairment in social, occupational, or other important areas of functioning predominate.  However, DO NOT meet the full criteria for any of the disorders in the feeding and eating disorders diagnostic class.  Used when the clinician chooses not to specify the reason that criteria are not met for a specific feeding and eating disorder.  This includes times when there is insufficient information to make a more specific diagnosis (e.g., in emergency room setting). | An eating disorder that does not meet the criteria for anorexia nervosa or bulimia nervosa. Individuals usually fall into one of three groups: sub-threshold symptoms of anorexia nervosa or bulimia nervosa, mixed features of both disorders or extremely atypical eating behaviours that are not characterized by either of the other established disorders.  Inclusion Terms:  • Atypical anorexia nervosa  • Atypical bulimia nervosa |
